# Interfacial Bond-Slip Model for CFRP Plate Externally Bonded to Corroded Steel Plate

**DOI:** 10.3390/ma15238488

**Published:** 2022-11-28

**Authors:** Anbang Li, Shanhua Xu

**Affiliations:** 1 School of Civil Engineering, Xi’an University of Architecture & Technology, Xi’an 710055, China; 2 State Key Laboratory of Green Building in Western China, Xi’an 710055, China

**Keywords:** bond-slip model, CFRP-to-steel, corrosion, bonding interface, double-lap joint

## Abstract

The purpose of this study is to establish the interfacial bond-slip model for CFRP plate externally bonded to corroded steel plate. The present bond-slip models for CFRP materials bonded to uncorroded steel plate were first reviewed. Thirty-four double-lap joints were tested to investigate the effect of corrosion duration and adhesive thickness on the bond behavior between CFRP plates and corroded steel plates, and the bond-slip curves for the bonding interface with different adhesive thickness and corrosion duration were obtained combined with the CFRP plate strain distribution data. A new bond-slip model for CFRP plate externally bonded to corroded steel plate was proposed, and the expression of the characteristic parameters, which included the maximum bond resistance $\tau_{f}$, the relative slip at the peak bond stress $s_{1}$, the fitting parameter *α*, and the interfacial fracture energy $G_{f}$, were also developed based on the careful regression analysis of the present data. The influence of the corrosion duration and construction adhesive thickness on the bond-slip relationship were accounted together and expressed as a new parameter; that is, the effective adhesive thickness $t_{\mathrm{eff}}$. The comparison between the predicted values and experimental results indicated that the proposed bond-slip model can be applied to reproduce the structural response of the CFRP plate-corroded steel plate double-lap joint with reasonable accuracy. The outcome of this study can provide meaningful references and essential data for the reliable application of CFRP strengthening systems in the performance improvement of corroded steel structures.

## 1. Introduction

Carbon fiber reinforced polymer (CFRP) materials, which possess the significant advantages of high strength/weight ratio, as well as excellent fatigue and corrosion resistance, have been widely applied in the field of concrete structure reinforcement in the past few decades. Recently, the strengthening of steel structures externally bonded with CFRP materials has attracted much attention, and the effectiveness of externally bonding CFRP to strengthen the bearing capacity [1,2,3], the fatigue behavior [4,5,6], and the stability performance [7,8,9] of steel structures have been verified by research results. Compared with other traditional repair and reinforcement methods, such as welding, bolting, or riveting [10,11], the strengthening system with CFRP material externally bonded to steel substrate presents the advantages of being highly efficient with minimized additional permanent load, eliminated stress concentration, and higher durability, etc. The bonding interface between the CFRP material and the steel substrate is the weakest position of the CFRP strengthened steel structure, and failure of the strengthening system usually starts from the failure of the bonding interface [12,13,14]; the quantitative description of the interfacial bond property is the premise of the performance evaluation of CFRP-strengthened steel structures [15,16]. The bond-slip relationship is the most important component of the bond performance, which essentially determines the interfacial shear stress distribution, the effective bond length of CFRP, and the ultimate bearing capacity of the bonding interface. An accurate bond-slip mode for CFRP externally bonded to steel substrate is the basis for the performance analysis of steel structures strengthened with CFRP materials.

Extensive experimental works have been conducted to investigate the bond performance between the CFRP material and the steel substrate, the effect of material properties of the CFRP [17,18,19] and the adhesive [20,21,22], the thickness of the adhesive layer [20,21,23], and surface preparation methods of steel plate [24,25], etc. These studies focused on primary bond characteristics, including failure mode, interfacial shear stress distribution, effective bond length, and ultimate load. Seven different kinds of bond-slip models have been developed for the CFRP-steel bonding interface, by Xia and Teng [21], Fernando [22], Dehghani et al. [26], Fawzia et al. [27], He and Xian [28], Wang and Wu [23], and Pang et al. [29], based on their respective test results. From the perspective of model structure and expressions of characteristic parameters, most of the existing bond-slip models have been developed based on appropriate simplification and regression analyses of experimental data using single-lap or double-lap test methods. According to the material types of the CFRP and the adhesive, as well as the surface treatment methods and the final interfacial failure modes involved in these experiments, in general, the bond-slip model for CFRP material externally bonded to steel substrate have been systematically investigated.

However, what should be noted is that almost all of the existing bond-slip models have been developed based on a default assumption that the steel substrate is flat and intact. For the existing steel structures that may need to be strengthened with CFRP, generally, they have been in service for a long time, and it is difficult to avoid corrosion on the surface of steel structures [30,31,32]. Limited experimental data have shown that corrosion damage would not only form the uneven rust pits on the steel substrate, but also change the surface roughness, contact area, and surface free energy of the steel substrate, which would inevitably affect the bonding performance between the CFRP material and the steel substrate [33,34,35]. To our knowledge, however, the effect of corrosion on the steel surface property and the bond-slip relationship between CFRP and steel plate have not been considered in the existing models; there have been no studies executed to develop the bond-slip model for CFRP material externally bonded to corroded steel plate.

The purpose of this study is to establish the interfacial bond-slip model for CFRP plate externally bonded to corroded steel plate. The existing bond-slip models for the CFRP-uncorroded steel interface were first reviewed. The experimental study investigating the bond behavior between CFRP plate and corroded steel plate was carried out by the authors and was then summarized, and the test data relevant to developing the bond-slip model were carefully analyzed. A new bond-slip model for CFRP plate externally bonded to corroded steel plate was then proposed based on the collected data. The accuracy of the proposed bond-slip model was finally verified by comparing the predicted and tested structural response of the CFRP plate-corroded steel plate double-lap joints. The outcome of this study can provide meaningful references and essential data for the reliable application of CFRP strengthening systems in the performance improvement of corroded steel structures.

## 2. Existing Bond-Slip Models for CFRP-Uncorroded Steel Interface

**Figure 1:**
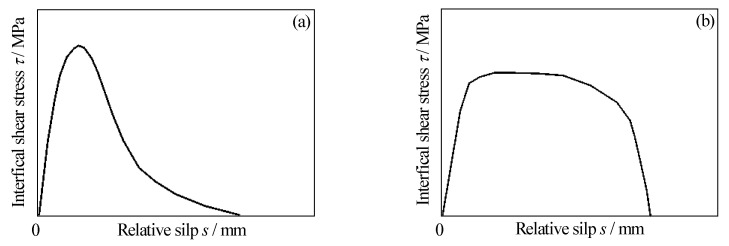
Two typical bond-slip curves of the bonding interface between CFRP materials and uncorroded steel substrates: (**a**) two-segment bond-slip curves; (**b**) three-segment bond-slip curves.

Several studies have been concerned with the bond behavior between CFRP materials and steel substrates. From the perspective of test results, the bond-slip curves of CFRP materials externally bonded to un-corroded steel plates have generally been presented as two typical forms, namely, two segment curves and three segment curves, as shown in Figure 1a,b, respectively. The two-segment bond-slip curve was mainly composed of the ascending and descending segments, and the bond-slip relationship could be approximated by a triangular shape (see Figure 1a). The interfacial shear stress between CFRP materials and steel substrates first increased and then decreased with the increase in slip value, and the obvious peak stress points and softening failure points were found on the bond-slip curves. The three-segment bond-slip curves were mainly composed of an ascending section, a platform section, and a descending section, and the bond-slip relationship could be approximated by a trapezoidal shape, as shown in Figure 1b. With the increase of the slip value, the interfacial shear stress first increased to the peak value, then remained unchanged, and finally decreased to failure. There were three characteristic points on the bond slip curve, namely, the starting point of the platform segment, the ending point of the platform segment, and the softening failure point. The main factors that determine the shape characteristics of the bond-slip curves of CFRP materials externally bonded to uncorroded steel substrates are the material properties of CFRP and adhesive, and the surface preparation methods for the steel substrates. The two-segment bond-slip curves mainly appeared on the bonding interfaces between CFRP plate (sheet) and steel substrates with linear adhesives [21,22,23,27,36], and the bonding interfaces between CFRP sheet and steel substrates with non-linear adhesives [22], whereas the three-segment bond-slip curves mainly appeared on the bonding interface between CFRP plates and steel substrates with non-linear adhesives [22,23,36].

As the bond-slip curves of the CFRP-steel bonding interface have been recorded as presenting different forms, several different types of bond-slip models [21,23,26,27,29] have been proposed by different researchers based on their respective test results. From the perspective of the structures, in terms of model expression, these models can be divided into four categories, namely, a bilinear model, a bi-curve model, a trilinear model, and a continuous model, as expressed in Equations (1)–(4), respectively. The bilinear model, bi-curve model, and continuous model have been regularly adopted to describe the aforementioned two-segment bond-slip curves, while the trilinear model has been adopted to describe the aforementioned three-segment bond-slip curves.

Bilinear bond-slip model [21,22,23,27,29]:

(1) $$\tau =\left\{ \begin{array}{l} \tau_{f}\frac{s}{s_{1}},\;s\leq s_{1}\\ \tau_{f}\frac{s_{f}-s}{s_{f}-s_{1}},\;s_{1}<s\leq s_{f}\\ 0,\;s>s_{f} \end{array}\right.$$

Bi-curve bond-slip model [22]:

(2) $$\tau =\left\{ \begin{array}{l} \tau_{f}\sqrt{\frac{s}{s_{1}}},\;s\leq s_{1}\\ \tau_{f}\exp\left(-\alpha \left(\frac{s}{s_{1}}-1\right)\right),\;s>s_{1} \end{array}\right.$$

Trilinear bond-slip model [22,23,26,36]:

(3) $$\tau =\left\{ \begin{array}{l} \tau_{f}\frac{s}{s_{1}},\;s\leq s_{1}\\ \tau_{f},s_{1}<s\leq s_{2}\\ \tau_{f}\frac{s_{f}-s}{s_{f}-s_{2}},\;s_{2}<s\leq s_{f}\\ 0,\;s>s_{f} \end{array}\right.$$

Continuous bond-slip model [36]:

(4) $$\tau =Ae^{-Bs}\left(1-e^{-Bs}\right)$$

where τ is the interfacial shear stress, *s* is the relative slip, $\tau_{f}$ is the maximum bond resistance, $s_{1}$ is the relative slip corresponding to the peak interfacial shear stress, $s_{2}$ is the relative slip at the end of the platform section, $s_{f}$ is the maximum relative slip. 

Table 1 presents a summary of the bond-slip models for CFRP materials bonded to uncorroded steel plate from previous studies; where *t*_a_ is the adhesive thickness, *E*_a_ and *G*_a_ are the elastic modulus and shear modulus of adhesives, respectively. *G*_f_ is the fracture energy of the bonding interfaces, *f*_t,a_ is the tensile strength of the adhesive, *w*_a_ is the tensile strain energy of the adhesive, *w*_c_ is the interlaminar shear energy dissipation of the CFRP plate. Furthermore, the material properties of the CFRP and the adhesive, the surface preparation methods for steel plate, and the interfacial failure modes corresponding to each model, are also presented in Table 1. 

It can be seen from Table 1 that, although the models proposed by different researchers have been similar in terms of the structural form of functions, the expressions of model parameters (such as peak shear stress, relative slip at peak shear stress, fracture energy, maximum slip, etc.) are quite different. The proposed models are basically empirical models based on regression analysis of the test results; different material properties and surface preparation methods will inevitably lead to different characteristic values of the bond-slip relationship. They indicate that when the bond-slip model for the CFRP materials externally bonded to uncorroded steel substrates is adopted for evaluating the bearing capacity of CFRP-strengthened steel structures, care must be taken regarding the applicable conditions of the selected model.

## 3. Summary of a New Experimental Study

In the present study, in order to investigate the interfacial bond behavior of CFRP plate externally bonded to corroded steel substrate, a series of double-lap tensile tests of CFRP-plates-corroded steel-plate-bonded joints were created [34,37]. Six kinds of corrosion duration and four kinds of adhesive thickness were the main factors considered. The bond characteristics, including failure modes, ultimate load, interfacial shear stress distribution, and effective bond length of the double-lap joint specimens, were tested and analyzed together with the effect of corrosion duration on the surface topography and roughness, as well as the surface free energy of the corroded steel plates. Test data relevant to establishing the bond-slip model are summarized in this section.

The corroded steel plates were cut from the flanges of hot-rolled Q235 H 350 × 175 × 7 × 11 beams with corrosion durations of 0, 3, 4, 6, 8, and 12 months, respectively. Unidirectional carbon-fiber-reinforced plastics (CFRP) plate “CFP-1-514” with a width and thickness of 50 mm and 1.4 mm, respectively, was adopted. Thixotropic and solventless two-part epoxy Sikadur-30CN was selected as the structural adhesive for bonding CFRP plates to corroded steel substrates. The mechanical properties of the adhesive and the steel plate were obtained by uniaxial tensile coupon tests based on the Chinese codes GB/T2567-2008 [38] and GB/T228.1-2010 [39], respectively. The main mechanical properties of all the materials are presented in Table 2.

**Figure 2:**
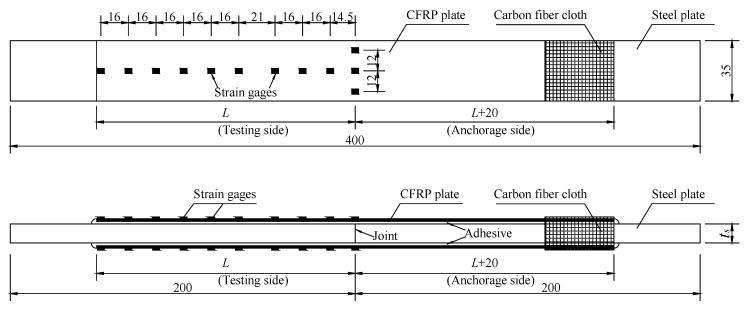
Schematic view of the double-lap joint (mm).

**Figure 3:**
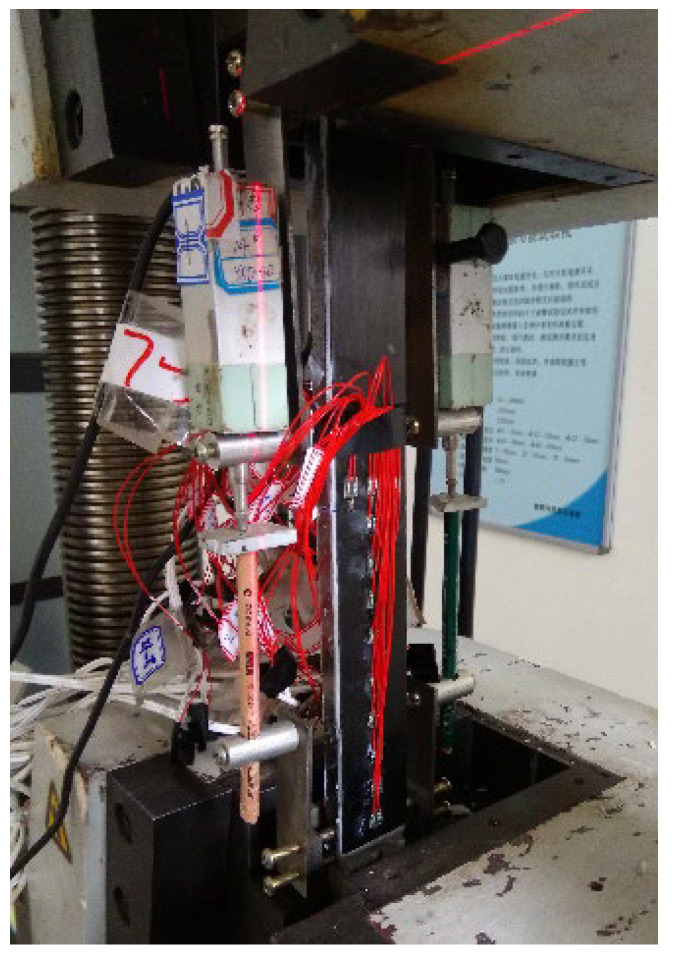
Test setup and instrumentation for the double-lap joint tensile tests.

A total of thirty-four double-lap joints were fabricated to investigate the bond properties between the CFRP plates and the corroded steel plates. Six levels of corrosion damage (0, 3, 4, 6, 8, and 12 months) for steel plates, four kinds of adhesive thickness (0.5, 1.0, 1.5, and 2.0 mm), and five kinds of bond length (30, 50, 80, 120, and 150 mm) for CFRP plates were used in the experiment. The name of the specimens consisted of three parts, and each part started with a letter and was followed with a number: C is corrosion durations (0, 3, 4, 6, 8, and 12 months); B is bond length (30, 50, 80, 120, and 150 mm); and T is the intended thickness of the adhesive layers (0.5, 1.0, 1.5, and 2.0 mm). Taking specimen C6-B5-T2 as an example, the corrosion duration of the steel plate, the bond length of CFRP plate, and the intended adhesive thickness were 6 months, 150 mm, and 1.0 mm, respectively. The double-lap joint was fabricated with two CFRP plates bonded to two corroded steel plates, the dimensions of the CFRP plates and the corroded steel plates were (2*L* + 20) × 35 × 1.4 mm and 200 × 35 × *t*_c_ mm (length × width × thickness), respectively, as shown in Figure 2. Where *L* is the intended bond length of the CFRP plate for the testing side of specimen, and the bond length of the anchorage side of the specimen is larger than *L* by 20 mm, to make sure failure always occurs on the testing side; *t*_c_ is the thickness of the corroded steel plates. The corroded steel plates were cut from the flanges of the corroded H beams by wire-cut electrical discharge machining (WEDM), and the corrosion products were carefully removed using an electric steel wire brush. Before fabricating, the surfaces of corroded steel plates and CFRP plates were cleaned using anhydrous alcohol to remove dust and greasy dirt, and surface characteristic tests, which include surface profile measurements and static contact angle measurements, were conducted to determine the effect of corrosion on the surface properties of steel substrates. A displacement-controlled step with a loading rate of 0.5 mm/min was carried out for the double-lap tensile tests. The interfacial bond behavior for the CFRP plate externally bonded to the corroded steel plate can be deduced by the strain distribution of the CFRP plate, which was recorded by a series of strain gauges pasted onto the CFRP plates, as shown in Figure 2 and Figure 3. More details of the experiments can be found in our other recent studies [34,37]. 

**Figure 4:**
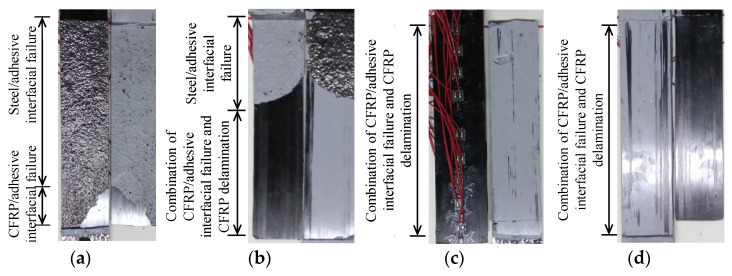
Effect of adhesive thickness on the failure modes for the specimens with the same corrosion duration. (**a**) C6-B5-T1 (back), (**b**) C6-B5-T2 (back), (**c**) C6-B5-T3 (front), (**d**) C6-B5-T4 (back).

Four kinds of failure modes, which included steel/adhesive interfacial debonding, cohesive failure, CFRP/adhesive interfacial debonding, and CFRP delamination, were observed in the experimental study. Test results showed that the failure mode of the bonding interface mainly depended on the adhesive thickness rather than the corrosion duration;. Moreover, corrosion was found to have a positive effect on the ultimate load for the double-lap joints with the same failure mode of steel/adhesive interfacial failure, and the effective bond length of the corroded specimens were obviously larger than that of the un-corroded ones. Results also indicated that, for the specimens with the same corrosion duration, the failure modes changed from the combination of steel/adhesive interfacial failure and CFRP/adhesive interfacial failure to the combination of CFRP/adhesive interfacial failure and CFRP delamination (see Figure 4); the ultimate load increased at first and decreased afterwards, and the effective bond length progressively increased with the adhesive thickness from 0.5 to 1.0, 1.5, and 2.0 mm.

**Figure 5:**
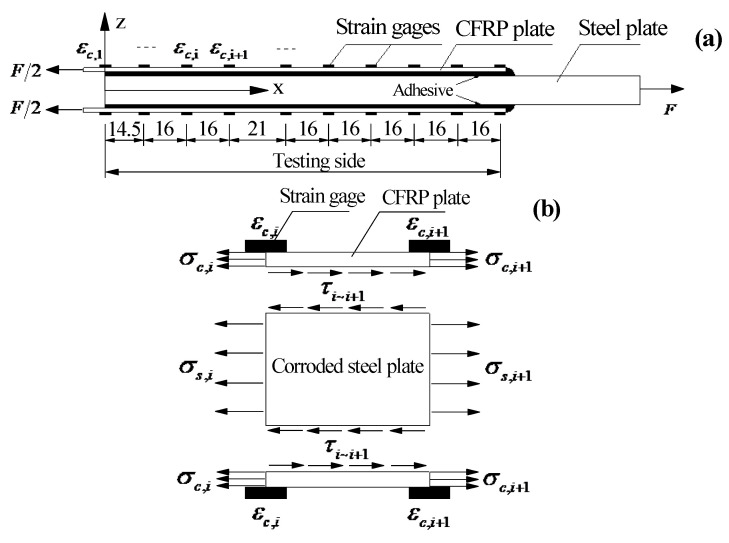
Schematic diagram for calculation of the interfacial shear stress and relative slip: (**a**) distribution of strain gauges on the surface of the CFRP plates on the testing side of the double-lap joint (mm); (**b**) microelement in the middle section of the adjacent strain gauges *i* and *i* + 1.

To determine the bond-slip relationship of the interface between the CFRP plate and the corroded steel plate, two physical quantities which include interfacial shear stress and relative slip need to be obtained. At present, the interfacial shear stress for the CFRP plate externally bonded to the steel substrate cannot be directly measured by technical means. The interfacial shear stress is indirectly calculated through the measurement of CFRP surface strain and the stress balance analysis of CFRP micro elements. The interfacial relative slip is mostly calculated through the integration of CFRP surface strain. The calculation process of interfacial shear stress and relative slip for the CFRP plate externally bonded to corroded steel plate is given in combination with the form of double-lap specimens, as shown in Figure 5.

According to the force balance condition of CFRP microelements, the interfacial shear stress $\tau_{i,i+1}$ of the bond interface between the corresponding positions of the adjacent strain gauges *i* and *i* + 1 can be expressed as:

(5) $$\tau_{i∼i+1}=\frac{\left(\sigma_{c,i}-\sigma_{c,i+1}\right)t_{c}}{L_{i∼i+1}}=\frac{\left(\varepsilon_{c,i}-\varepsilon_{c,i+1}\right)E_{c}t_{c}}{L_{i∼i+1}}$$

where $\sigma_{c,i}$ and $\sigma_{c,i+1}$ are the tensile stress of the CFRP plate at the corresponding positions of strain gauges *i* and *i* + 1, respectively; $\varepsilon_{c,i}$ and $\varepsilon_{c,i+1}$ are the tensile strain of the CFRP plate at the corresponding positions of strain gauges *i* and *i* + 1, respectively; $E_{c}$ and $t_{c}$ are the elastic modulus and thickness of CFRP plates, respectively; $L_{i∼i+1}$ is the distance between the adjacent strain gauges *i* and *i* + 1.

The interfacial relative slip $s_{i∼i+1}$ of the bonding interface between the corresponding positions of the adjacent strain gauges *i* and *i* + 1 are equal to the difference between the tensile deformation of the CFRP plate and the corroded steel plate in the corresponding interval, i.e.,

(6) $$s_{i∼i+1}=\Delta_{c,i∼i+1}-\Delta_{s,i∼i+1}=\frac{\left(\varepsilon_{c,i}+\varepsilon_{c,i+1}\right)L_{i∼i+1}}{2}-\frac{\left(\varepsilon_{s,i}+\varepsilon_{s,i+1}\right)L_{i∼i+1}}{2}$$

where $\Delta_{c,i∼i+1}$ and $\Delta_{s,i∼i+1}$ are the tensile deformation of the CFRP plate and corroded steel plate between the corresponding positions of the adjacent strain gauges *i* and *i* + 1, respectively, $\varepsilon_{s,i}$ and $\varepsilon_{s,i+1}$ are the tensile strain of the steel plate at the corresponding positions of strain gauges *i* and *i* + 1, respectively. 

By intercepting the calculation unit from the joint of the double-lap specimen to the corresponding position of strain gauge *i*, the following formula can be obtained according to the force balance condition of the calculation unit:

(7) $$2\varepsilon_{c,1}E_{c}t_{c}+\sigma_{s,1}t_{s}=2\varepsilon_{c,i}E_{c}t_{c}+\varepsilon_{s,i}E_{s}t_{s}$$

where $\varepsilon_{c,1}$ is the tensile strain of the CFRP plate at the corresponding position of the double-lap joint (shown in Figure 5a), $E_{s}$ and $t_{s}$ are the elastic modulus and average thickness of the corroded steel plate, $t_{s}=\left(1-\rho \right)t_{0}$, ρ is the weight loss rate of the corroded steel plate, $t_{0}$ is the thickness of the un-corroded steel plate, $\sigma_{s,1}$ is the tensile stress of the corroded steel plate at the corresponding positions of the double-lap joint. According to the stress boundary condition at the corresponding positions of the double-lap joint, $\sigma_{s,1}=0$, Equation (7) can be rewritten as the following:

(8) $$\varepsilon_{s,i}=\frac{2\left(\varepsilon_{c,1}-\varepsilon_{c,i}\right)E_{c}t_{c}}{E_{s}t_{s}}$$

Substitute Equation (8) into Equation (6), which yields

(9) $$s_{i∼i+1}=\left(\frac{1}{2}+\frac{E_{c}t_{c}}{E_{s}t_{s}}\right)\left(\varepsilon_{c,i}+\varepsilon_{c,i+1}\right)L_{i∼i+1}-\frac{2\varepsilon_{c,1}E_{c}t_{c}}{E_{s}t_{s}}L_{i∼i+1}$$

**Figure 6:**
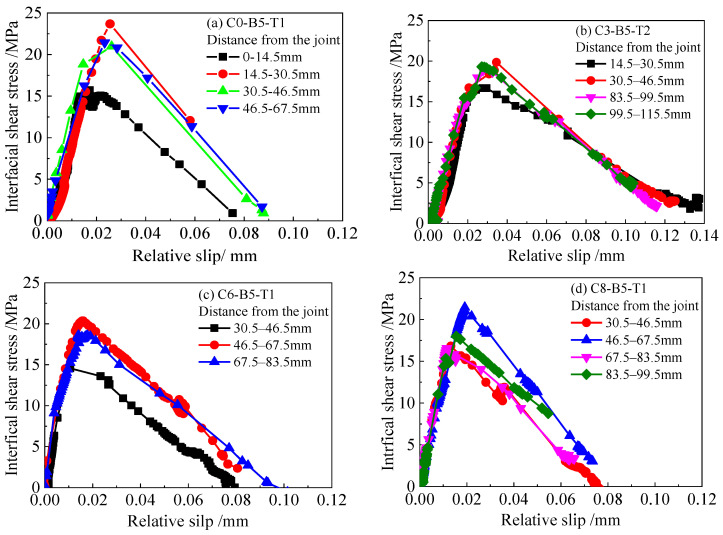
Typical bond–slip relationships for the CFRP plate externally bonded to the corroded steel plate with different corrosion durations and adhesive thicknesses: (**a**) specimen C0-B5-T1, (**b**) specimen C3-B5-T2, (**c**) specimen C6-B5-T1, and (**d**) specimen C8-B5-T1.

According to Equations (5)–(9), the bond-slip curves for the CFRP plate externally bonded to the corroded steel plate can be obtained combined with the data of strain distribution on the surface of the CFRP plates obtained from the previous experiment. Figure 6 presents typical bond–slip relationships for the CFRP plate externally bonded to corroded steel plate with different corrosion durations and adhesive thicknesses. As presented in Figure 6, the shape of the bond-slip curves, which are calculated based on the strain distribution at different locations, is similar and approximately triangular. The bond-slip curves present typical two-segment characteristics; i.e., with the increase of the relative slip value of the interface, the interface shear stress increases at first and then decreases afterwards. The slope of the ascending segment curves decreases with the increase in the slip value, indicating that a certain plastic deformation occurs at the bonding interface between the CFRP plate and the corroded steel plate before the interfacial shear stress reaches the peak value. Furthermore, the dispersion of the descending segment of the curves is more significant than that of the ascending segment, which may be related to the brittle characteristics of the interface failure.

## 4. Development of the Bond-Slip Model

The summary of the previous bond-slip models for CFRP materials bonded to uncorroded steel plate, as presented in Section 2, have shown that although similar models have been adopted by different researchers to describe the bond-slip relationship of the interface between CFRP materials and uncorroded steel substrate, the model parameters greatly vary. Different material properties of the CFRP plate (sheet) and the adhesive, as well as surface preparation methods, will inevitably lead to different characteristic values of the bond-slip relationship. Surface characteristic changes caused by corrosion are significantly different from the existing treatment measures in terms of both nature and degree. Therefore, it is necessary to develop a new bond-slip model for the CFRP plate externally bonded to a corroded steel plate. 

**Figure 7:**
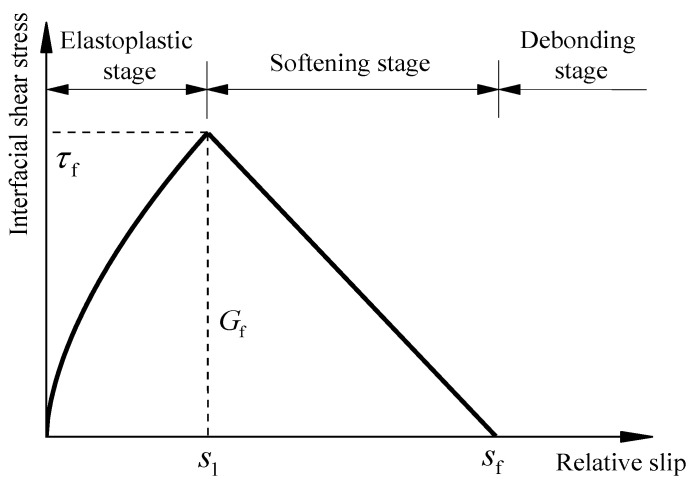
Bond–slip model for CFRP plate and corroded steel plate.

According to the characteristics of bond-slip curves observed in Section 3, and referring to existing bond-slip models of the bond interface between the CFRP material and the uncorroded steel plate, a new bond-slip model for CFRP plate externally bonded to corroded steel plate is proposed, as shown in Figure 7. The model is composed of three stages, namely, the elastoplastic stage, the softening stage, and the debonding stage, as expressed in Equation (10). In the elastoplastic stage, the power function is adopted to describe the physical phenomenon that the interfacial bond stiffness corresponding to the ascending segment of the bond-slip curves decreases with the increase in the slip value, and the linear function is applied to describe the softening stage, as the relative slip value increases from $s_{1}$ to $s_{f}$, the corresponding interfacial shear stress gradually decreases from $\tau_{f}$ to 0.

(10) $$\tau =\left\{ \begin{array}{l} \tau_{f}{\left(\frac{s}{s_{1}}\right)}^{\alpha },\;s\leq s_{1}\\ \tau_{f}\frac{s_{f}-s}{s_{f}-s_{1}},\;s_{1}<s\leq s_{f}\\ 0,\;s>s_{f} \end{array}\right.$$

where τ is the interfacial shear stress, *s* is the relative slip, $\tau_{f}$ is the maximum bond resistance, $s_{1}$ is the relative slip corresponding to the peak interfacial shear stress, *α* is the fitting parameter for the ascending segment of the bond-slip curve, $s_{f}$ is the maximum relative slip. Notably, the descending segments of the bond-slip curves, which are calculated based on the strain distribution data at different positions of the CFRP plate, present a certain degree of dispersion. The maximum relative slip value of the interface $s_{f}$ is determined according to the principle of equal fracture energy of the interface. $s_{f}$ can be calculated according to the following equations:

(11) $$G_{f}=\int_{0}^{s_{1}}\tau_{f}{\left(s/s_{1}\right)}^{\alpha }+\frac{1}{2}\tau_{f}\left(s_{f}-s_{1}\right)=\frac{1}{2}\tau_{f}s_{f}+\frac{1-\alpha }{2\left(1+\alpha \right)}\tau_{f}s_{1}$$

(12) $$s_{f}=\frac{2G_{f}}{\tau_{f}}-\frac{1-\alpha }{1+\alpha }s_{1}$$

where $\tau_{f}$ and $s_{1}$ are directly obtained from experimental results, *α* is obtained by fitting test data, $G_{f}$ is the fracture energy of the bonding interfaces which can be obtained by integration of the bond-slip curves. Table 3 presents the characteristic value of bond properties, which include the maximum bond resistance $\tau_{f}$, the relative slip corresponding to the peak interfacial shear stress $s_{1}$, the fitting parameter *α*, the fracture energy $G_{f}$ and the maximum relative slip $s_{f}$, for the tested specimens with a CFRP bond length of 150 mm. 

### 4.1. Maximum Bond Resistance $\tau_{f}$

**Figure 8:**
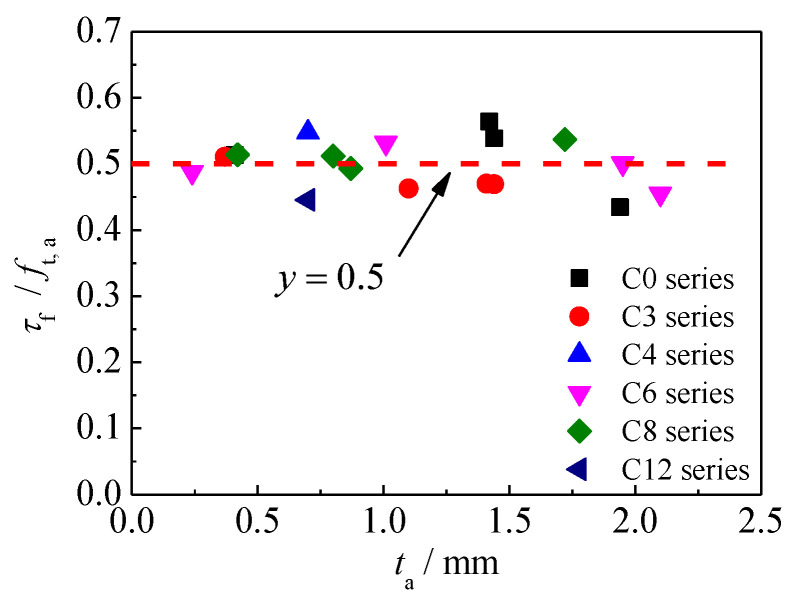
The ratio of maximum bond resistance to the tensile strength of the adhesive $\tau_{f}/f_{t,a}$ versus the adhesive thickness $t_{a}$ for the specimens with different corrosion duration.

The research results of previous studies [21,23,27] have shown that the maximum bond resistance $\tau_{f}$ of the bonding interface between the CFRP material and the uncorroded steel substrate was mainly affected by the strength of the adhesive, the surface characteristics of the steel plate, and the failure mode of the bonding interface, etc. Whereas, the thickness of the adhesive layer presented no significant effect on the maximum bond resistance, and the maximum bond resistance $\tau_{f}$ was always expressed as a function of the ultimate tensile strength of the adhesive $f_{t,a}$; see Table 2. Figure 8 presents the ratio of maximum bond resistance to the tensile strength of the adhesive $\tau_{f}/f_{t,a}$ versus the adhesive thickness $t_{a}$ for the specimens with different corrosion duration. As shown in Figure 8, the value of $\tau_{f}/f_{t,a}$ basically remains constant for the bonding interfaces with different corrosion durations and adhesive thicknesses. Regression analysis of all test data showed that the average value and the coefficient of variation of $\tau_{f}/f_{t,a}$ are 0.4993 and 0.075, respectively. Therefore, the maximum bond resistance $\tau_{f}$ can be expressed as:

(13) $$\tau_{f}=0.5f_{t,a}$$

where $f_{t,a}$ is the tensile strength of the adhesive.

### 4.2. Relative Slip at the Peak Bond Stress $s_{1}$

The relative slip of the bonding interface between the CFRP plate and the corroded steel plate consists of the shear deformations of the CFRP plate, adhesive layer, and the corroded steel plate. Considering that the shear modulus of the adhesive is far lower than that of the steel plate and the CFRP plate, the relative slip value of the bonding interface is mainly composed of the shear deformation of the adhesive layer; therefore, the relative slip at the peak bond stress $s_{1}$ is often described as a function of the thickness of the adhesive layer, the shear modulus, and the shear strength of the adhesive in various empirical models, as expressed in Table 1. What should be pointed out is that the surfaces of both the uncorroded steel plate and the corroded steel plate have certain levels of roughness, which is bound to contribute to the additional adhesive thickness of the bonding interface. The difference is that the surface roughness of the uncorroded steel plate is too small, so its contribution to the adhesive layer thickness is usually ignored. While as for the bonding interface between the CFRP plate and the corroded steel plate, the proportion of the additional adhesive thickness, which is caused by the rusted rough surface of the corroded steel plate, to the effective adhesive thickness is much higher than that of the uncorroded steel plate. The increase in effective adhesive thickness caused by corrosion even changes the failure mode of the bonding interface between the CFRP plate and the steel plate [34]. 

**Figure 9:**
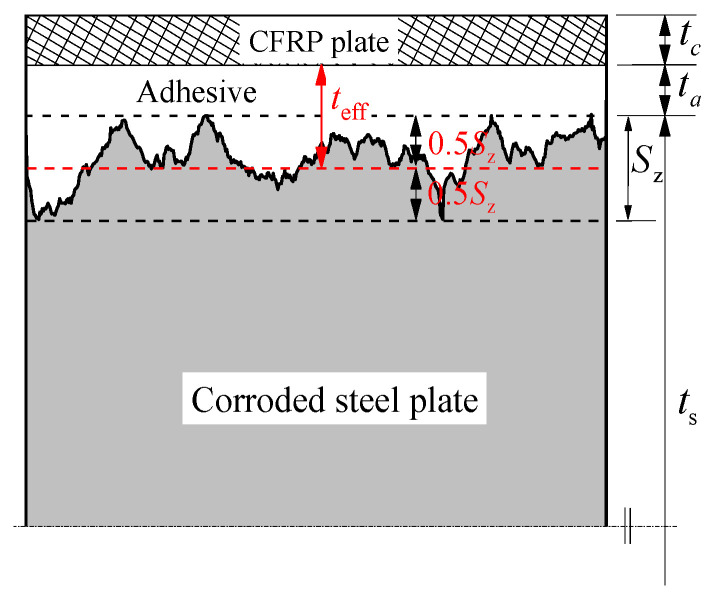
Schematic diagram of the cross-section for the bonding interface between the CFRP plate and the corroded steel plate.

Figure 9 presents the schematic diagram of the cross-section for the bonding interface between the CFRP plate and the corroded steel plate; where $t_{a}$ is the construction adhesive thickness, the maximum height $S_{z}$ of the contour surface is equal to the difference between the maximum depth of the corrosion pits and the maximum height of the edge of the corrosion pits on the corroded steel surface, which reflects the two extreme distributions of the corrosion surface roughness, and can be calculated by applying the following equation [40]:

(14) $$S_{z}=\underset{(x,y)\in A}{\max}\left[z(x,y)\right]-\underset{(x,y)\in A}{\min}\left[z(x,y)\right]$$

where *A* is the nominal area of the scanning surface, *z*(*x*, *y*) is the scanned height of the scale-limited surface at position (*x*, *y*). More details of the calculation process and surface scanning can be found in our recent study [34,37]. The values of *S*_z_ for the corroded steel plates with different corrosion durations are also listed in Table 3. The influence of the corrosion duration and construction adhesive thickness on the bond-slip relationship can be accounted together and expressed as a new parameter: the effective adhesive thickness $t_{\mathrm{eff}}$. As shown in Figure 9, the effective adhesive thickness $t_{\mathrm{eff}}$ of the bonding interface between the CFRP plate and the corroded steel plate can be approximately defined as the sum of the construction adhesive thickness $t_{a}$ and the additional adhesive thickness of 0.5 times the maximum height $S_{z}$, i.e.,

(15) $$t_{\mathrm{eff}}=t_{a}+0.5S_{z}$$

**Figure 10:**
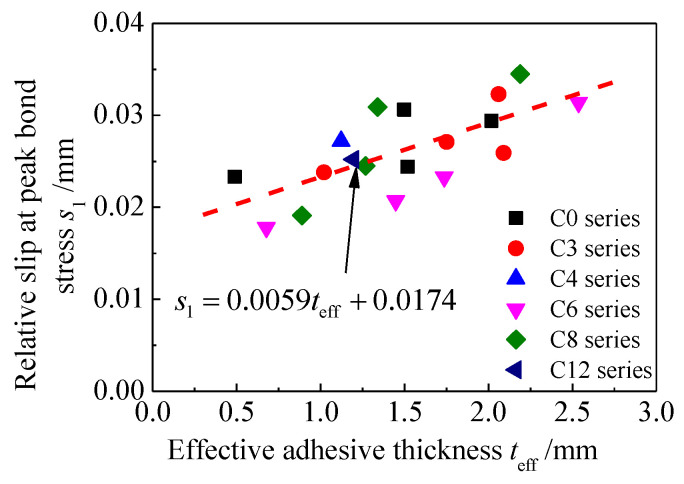
Transformation of relative slip at the peak bond stress $s_{1}$ with respect to effective adhesive thickness $t_{\mathrm{eff}}$.

Figure 10 illustrates the transformation of the relative slip at the peak bond stress $s_{1}$ with respect to the effective adhesive thickness $t_{\mathrm{eff}}$. As illustrated in Figure 10, $s_{1}$ is approximately positively correlated with $t_{\mathrm{eff}}$, which is similar to the variation rules found in studies on the interfacial bond behavior between CFRP plates and uncorroded steel plates [21,23,26,27]. The parameter expression of the relative slip at the peak bond stress $s_{1}$ is given by fitting the test data:

(16) $$s_{1}=0.0059t_{\mathrm{eff}}+0.0174$$

### 4.3. Fitting Parameter α

**Figure 11:**
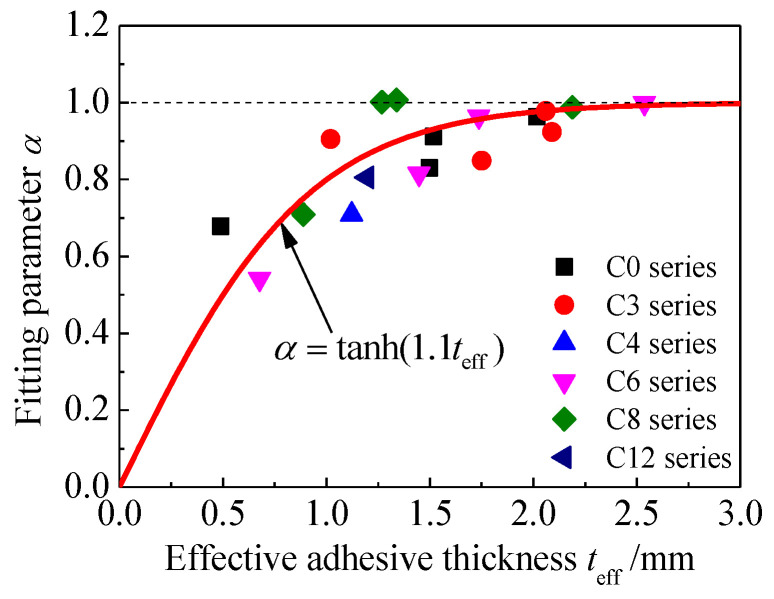
Fitting parameter *α* versus the effective adhesive thickness $t_{\mathrm{eff}}$.

It can be seen from Figure 6 that the ascending segments of the bond-slip curves present an upward convex shape; the slope of the bond-slip curves decreases with the increase of the slip value before the bond stress reaches the peak value. Here, the power function $y=x^{\alpha }$ (where $y=\tau /\tau_{f}$, $x=s/s_{1}$) is applied to describe the physical phenomenon that the interfacial bond stiffness decreases with the increase in the slip value in the ascending segment of the bond-slip relationship. In terms of the physical meaning of the model, with the increase in the slip value, the stiffness of the ascending segment of the bond-slip relationship will only gradually decrease, so the parameter *α* yields α≤1. Furthermore, from the perspective of the function characteristics of $y=x^{\alpha }\;(x\leq 1)$, when α is less than 1.0, the smaller the value of α, the more obvious the ascending curves. The value of α, which is obtained based on the data fitting for the specimens with different corrosion durations and adhesive thicknesses, is listed in Table 3. Figure 11 presents the fitting parameter *α* versus the effective adhesive thickness $t_{\mathrm{eff}}$. As shown in Figure 11, with the increase of the effective thickness of the adhesive layer, α shows a process of gradually increasing and finally converging to 1.0, which means that with the increase of the effective thickness of the adhesive layer, the degree of the upward convex of the ascending section of the bond-slip curves gradually decreases, and the elastic-plastic characteristics of the bond-slip curves gradually disappear before the interfacial bond stress reaches the peak value. The above changing process is consistent with the influence of the effective adhesive thickness on the interfacial failure mode of the double-lap specimens, i.e., the failure modes change from the combination of steel/adhesive interfacial failure and CFRP/adhesive interfacial failure to the combination of CFRP/adhesive interfacial failure and CFRP delamination, accompanied by a construction adhesive thickness increase from 0.5 mm to 2.0 mm; the brittleness of the interface failure becomes more and more obvious [34]. Here, according to the physical meaning and boundary conditions of the fitting parameter α, the function y = tanh(nx) is constructed to fit the scattered points in Figure 11 to obtain an expression of the parameter α:

(17) $$\alpha =\tanh(1.1t_{\mathrm{eff}})$$

### 4.4. Interfacial Fracture Energy $G_{f}$

**Figure 12:**
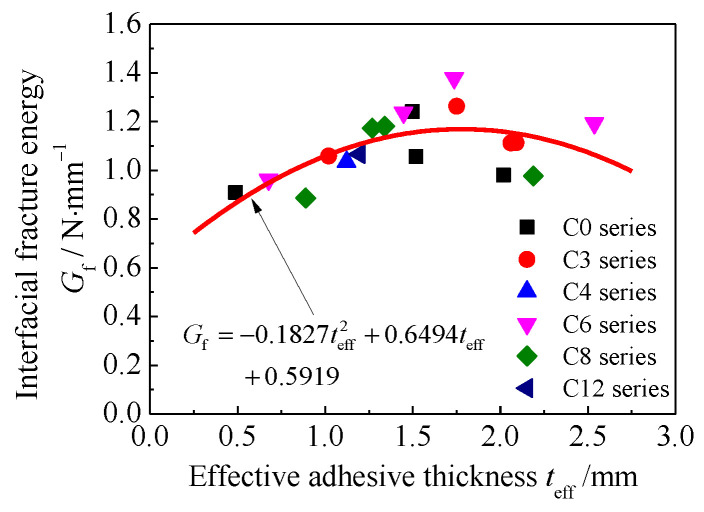
Interfacial fracture energy $G_{f}$ versus the effective adhesive thickness $t_{\mathrm{eff}}$.

The interfacial fracture energy can be obtained from the integration of the experimental bond-slip curves. The calculated interface fracture energy for all the specimens with a CFRP bond length of 150 mm on the testing side of the double-lap joint are presented in Table 3. Figure 12 shows the interfacial fracture energy versus the effective adhesive thickness for specimens with different corrosion durations. As shown in Figure 12, a similar trend was found for the interfacial fracture energy of the corresponding specimens with different corrosion durations; that is, with the increase of the effective adhesive thickness, the interfacial fracture energy increased at first and then resorted to a steady value, and finally decreased, which is also consistent with the transformation of the ultimate load of the double-lap joint specimens with respect to the adhesive thickness [34]. By comparing the existing bond-slip models of CFRP materials externally bonded to uncorroded steel substrate, as presented in Table 1, it can be found that the interface fracture energy was usually expressed as a function of the adhesive thickness and the tensile strain energy of the adhesive, or the interlaminar shear energy dissipation of the CFRP, which had a clear physical significance for the bonding interface, where only the cohesive failure in the adhesive layer or CFRP delamination occurred, which might not be suitable for describing the bonding interface with other failure modes, such as steel/adhesive interfacial debonding and CFRP/adhesive interfacial debonding. In this study, four failure modes which included steel/adhesive interfacial debonding, cohesive failure, CFRP/adhesive interfacial debonding, and CFRP delamination were observed for the bonding interfaces with different corrosion durations and adhesive thicknesses. To consider the influence of corrosion surface morphology and construction adhesive thickness on the interfacial bond-slip relationship for the CFRP plate externally bonded to the corroded steel substrate, and to facilitate engineering application. The interface fracture energy $G_{f}$ is expressed as a function of the effective adhesive thickness $t_{\mathrm{eff}}$ on the basis of regression analysis of test data:

(18) $$G_{f}=-0.1827t_{\mathrm{eff}}^{2}+0.6494t_{\mathrm{eff}}+0.5919$$

## 5. Validation of the Proposed Bond-Slip Model

The proposed interfacial bond-slip model is verified by numerical methods in this section. Finite element software (ANSYS^®^14.5) (ANSYS, Inc., Pittsburgh, PA, USA) is employed to conduct the numerical study, and the nonlinear spring element COMBIN39 is applied to simulate the interfacial bond-slip relationship to establish the finite element model of CFRP plate-corroded steel double-lap joints. In essence, the structural response, which includes the load-displacement relationship and the CFRP plate strain distribution for the double-lap joint specimens under tensile load, are determined by the local interfacial bond-slip relationship between the CFRP plate and the corroded steel plate. Hence, the accuracy of the proposed bond-slip model in this paper can be verified by comparing the load-displacement curves and strain distribution of the CFRP plates, which can be directly measured from the experimental tests.

### 5.1. Finite Element Models

**Figure 13:**
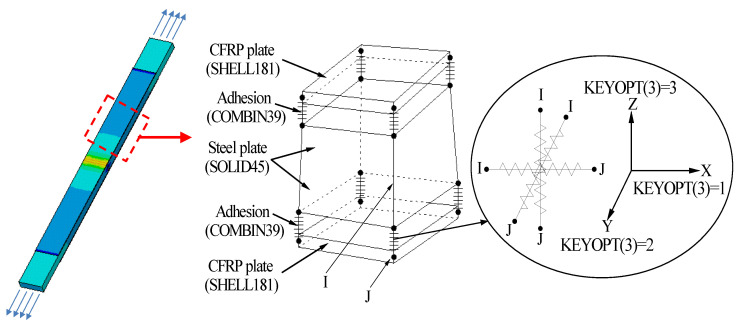
Element type and constraint relationships in the finite element models.

**Figure 14:**
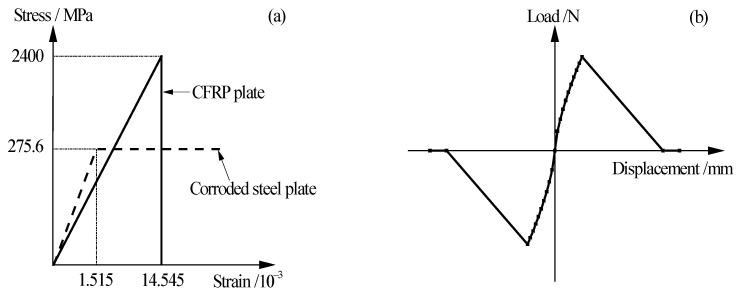
Material constitutive model in finite element models: (**a**) stress-strain relationship of the corroded steel plate and the CFRP plate, (**b**) real constant (*F*-*D* relationship) of spring element.

The CFRP plate-corroded steel double-lap joint specimen, which was tensile tested in our recent study [34,37], is adopted as the prototype structure by which to conduct the finite element analysis. The thickness of the corroded steel plate is taken as *t*_0_(1−*ξ*%); where *t*_0_ is the thickness of the uncorroded steel plate, *ξ* is the weight loss rate of the corroded steel plate. The thickness of the adhesive layer is determined according to Equation (15). Figure 13 presents the element type and constraint relationships in the finite element models. As shown in Figure 13, the corroded steel plate is simulated by a three-dimensional 8-node structural solid element, SOLID45, the CFRP plate is simulated by a 4-node finite strain shell element, SHELL181, and the adhesive layer between the CFRP plate and the corroded steel plate is simulated by nonlinear spring element COMBIN39; three mutually perpendicular elements of COMBIN39 are inserted between the element nodes of the corroded steel plate and the CFRP plate, and the degree of freedom constraints in the x, y, and z directions are achieved by rewriting the element characteristic parameters KEYPOT (3). Figure 14 illustrates the material constitutive models in the finite element model. As shown in Figure 14a, the ideal elastoplastic model with an elastic modulus of 181.9 GPa and a yield strength of 275.6 MPa is adopted for the corroded steel plate, and the linear elastic model with an elastic modulus of 165 GPa and an ultimate tensile strength of 2400 MPa is adopted for the CFRP plate. The real constant of the spring element COMBIN39 (i.e., the *F*-*D* curves, see Figure 14b), which reflect the bond-slip behavior of the bonding interface between CFRP plate and corroded steel plate, is calculated according to the interfacial bond-slip model of the CFRP plate externally bonded to the corroded steel plate, as proposed in Section 4:

(19) $$F=\tau a^{2},D=s$$

where *F* is the force of the spring element COMBIN39, *D* is the displacement between the nodes at both ends of the spring element, *τ* and *s* are the bond stress and slip, as expressed in Equation (10), $a^{2}$ is the area covered by individual springs.

### 5.2. Comparison between Finite Element Analysis Results and Experimental Results

**Figure 15:**
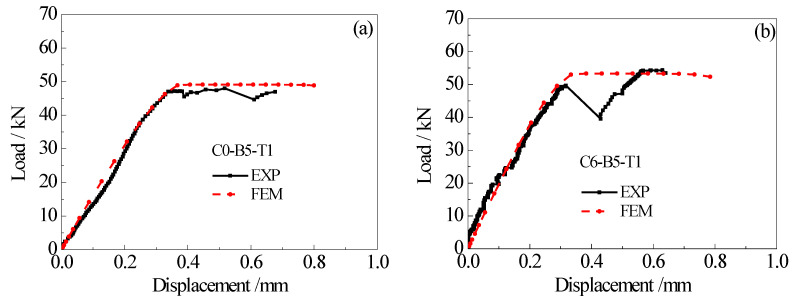
Comparison between the predicted and tested load-displacement curves: (**a**) specimen C0-B5-T1, (**b**) specimen C6-B5-T1.

**Figure 16:**
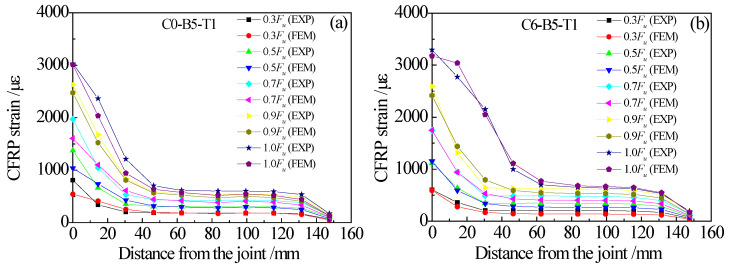
Comparison between the predicted and tested strain distribution of CFRP plates: (**a**) specimen C0-B5-T1, (**b**) specimen C6-B5-T1.

Taking the specimens C0-B5-T1 and C6-B5-T1 as examples, Figure 15 presents a comparison between the predicted and tested load-displacement curves. As shown in Figure 15, the finite element analysis results are in good agreement with the experimental results. Particularly, the predicted and tested load-displacement curves are almost identical before the load of the double-lap joint reaches the ultimate value. Although the platform section of the load-displacement curve measured in the test fluctuates to some extent due to the brittle failure characteristics of the specimen itself after reaching the ultimate load, which makes it slightly different from the finite element analysis result, the predicted results are still in good agreement with the experimental data in terms of the value of ultimate load. Figure 16 shows the comparison between the predicted and tested strain distribution of CFRP plates. Considering that the thickness of the adhesive layer in the finite element model is the mean value of the thickness of the front and back adhesive layers, the test results in Figure 16 are also the mean values of the measured strains of CFRP plates on both sides of the test piece. It can be seen from Figure 16 that the CFRP strain distribution for the double-lap joint specimens obtained from finite element analysis are highly consistent with the experimental data. It indicates that the interfacial bond-slip model proposed in this paper can be applied to reproduce the load-displacement relationship and CFRP plate strain distribution for the CFRP plate-corroded steel plate double-lap joint under tensile load with reasonable accuracy. 

## 6. Conclusions

In this study, the present bond-slip models for CFRP materials bonded to uncorroded steel plates were first reviewed. It showed that, although a similar model has been adopted by different researchers to describe the bond-slip relationship of the interface between CFRP materials and uncorroded steel substrate, the model parameters recommended by different researchers have greatly varied. Different material properties of the CFRP plate (sheet) and the adhesive, as well as surface preparation methods, will inevitably lead to different characteristic values of the bond-slip relationship. The recent experimental studies on the bond behavior between the CFRP plate and the corroded steel plate conducted by the authors of this paper were then summarized. The calculation method for the interfacial shear stress and the relative slip was first established, and the bond-slip curves for the bonding interface with different adhesive thicknesses and corrosion durations were obtained combined with the CFRP plate strain distribution data. A new bond-slip model for CFRP plates externally bonded to corroded steel plates was then proposed and numerical verification was carried out. The comparison between the predicted values and experimental results indicated that the proposed interfacial bond-slip model can be applied to reproduce the structural response of the CFRP plate-corroded steel plate double-lap joint with reasonable accuracy. Nevertheless, it is important to note that the proposed bond-slip model, as well as the implicated function and parameters, are only feasible for the local bond properties of CFRP plates externally bonded to corroded steel substrates. Furthermore, the quantitative rules established basing on the experimental data fitting are restricted to the CFRP plate with a normal elasticity modulus of 165 GPa, the linear elasticity adhesive of Sikadur-30CN, as well as the surface preparation method of electric steel wire brush and solvent cleaning. Additional experimental studies concerning other forms and modulus of CFRP materials, adhesive types, and surface preparation methods are still needed for enlarging of the application range and improvement of the accuracy of the models. The outcome of this study can provide meaningful references and essential data for the reliable application of CFRP strengthening systems in the performance improvement of corroded steel structures.

## Figures and Tables

**Table 1:** Summary of bond-slip models for CFRP materials bonded to uncorroded steel plate.

Refs.	Models	Parameter Expression	Material Properties of CFRP and Adhesive	Surface Preparation Methods	Failure Modes
Xia and Teng, 2005 [21]	Equation (1)	$\begin{array}{l} \tau_{f}=0.8f_{t,a};\;s_{1}=\tau_{f}t_{a}/G_{a};\;\\ s_{f}=2G_{f}/\tau_{f};\;\\ G_{f}=31{\left(f_{t,a}/G_{a}\right)}^{0.56}t_{a}{}^{0.27} \end{array}$	CFRP plate ($E_{c}=165\mathrm{GPa}$); adhesive A, B, and C	The surfaces of steel plates were sandblasted and cleaned with acetone	Cohesive failure
Fawzia, 2010 [27]	Equation (1)	$\begin{array}{l} \tau_{f}=f_{t,a};\;\\ s_{1}=t_{a}/10;\\ s_{f}=\left\{ \begin{array}{l} t_{a}/4,t_{a}=0.1∼0.5\mathrm{mm}\\ 0.125+\left(t_{a}-0.5\right)/10,\;t_{a}=0.5∼1\mathrm{mm} \end{array}\right. \end{array}$	CFRP sheet ($E_{c}=640;240\mathrm{GPa}$); linear and non-linear adhesives Araldite 420, MBrace, and Sikadur 30	The surfaces of steel plates were sandblasted and cleaned with acetone	Steel/adhesive interface debonding and CFRP delamination
Fernando, 2010 [22]	Equation (1)	$\begin{array}{l} \tau_{f}=0.9f_{t,a};\;s_{1}=0.3\tau_{f}{\left(t_{a}/G_{a}\right)}^{0.65};\;\\ s_{f}=2G_{f}/\tau_{f};G_{f}=628t_{a}{}^{0.25}w_{a}{}^{2} \end{array}$	CFRP plate ($E_{c}=150;235;340\mathrm{GPa}$); linear adhesives Sika 30 and Sika 330	The top surfaces of the steel plates were solvent-wiped, grit-blasted using 0.25 mm angular grit, and then further cleaned using a vacuum head	Cohesive failure; Delamination within CFRP plate
	Equation (2)	$\begin{array}{l} \tau_{f}=0.9f_{t,a};\;s_{1}=0.3\tau_{f}{\left(t_{a}/G_{a}\right)}^{0.65};\;\\ s_{f}=2G_{f}/\tau_{f};\alpha =\frac{3\tau_{f}s_{1}}{3G_{f}-2\tau_{f}s_{1}};\;\\ G_{f}=628t_{a}{}^{0.25}w_{a}{}^{2} \end{array}$			
	Equation (3)	$\begin{array}{l} \tau_{f}=0.9f_{t,a};\;s_{1}=0.081\mathrm{mm};\;\\ s_{2}=0.8\mathrm{mm};\\ s_{f}=2\left(G_{f}-\tau_{f}\left(s_{2}-0.5s_{1}\right)\right)/\tau_{f}+s_{2} \end{array}$	CFRP plate ($E_{c}=150;235;340\mathrm{GPa}$); non-linear adhesives Araldite 2015 and Araldite 420		
Dehghani, 2012 [26]	Equation (3)	$\begin{array}{l} \tau_{f}=0.8f_{t,a};\;s_{1}=\tau_{f}t_{a}/G_{a}\\ s_{1}=\frac{s_{f}}{3};\;s_{f}=\frac{3G_{f}}{2\tau_{f}}+\frac{3}{4}s_{1} \end{array}$	The bond-slip model was proposed by adding a plastic part to the conventional bilinear model. Analysis of the bonded connection was performed by simulation of the plate and adhesive in a new form of equivalent springs.		
He and Xian, 2016 [36]	Equation (3)	$\begin{array}{l} \tau_{f}=0.5f_{t,a};s_{1}=0.08\mathrm{mm};\\ s_{2}=G_{f}/\tau_{f};s_{f}=s_{1}+s_{2};\\ G_{f}=10.65t_{a}^{1.745}w_{a}{}^{0.437} \end{array}$	CFRP plate ($E_{c}=185\mathrm{GPa}$); non-linear adhesive T_C_	The surfaces of steel plates were de-rusted by abrasive paper and cleaned with acetone firstly and then treated by 0.1 mm alumina grit	Delamination within CFRP plate
	Equation (4)	$\begin{array}{l} \tau_{f}=0.5f_{t,a};\;A=4\tau_{f};\;\\ B=2\tau_{f}/G_{f};\\ G_{f}=10.65t_{a}^{1.745}w_{a}{}^{0.437}; \end{array}$	CFRP plate ($E_{c}=185\mathrm{GPa}$); linear adhesives T_1_ and T_S_		Cohesive failure
Wang and Wu, 2018 [23]	Equation (1)	$\begin{array}{l} \tau_{f}=0.9f_{t,a};\;s_{1}=2.9\frac{\tau_{f}}{G_{a}}t_{a}^{0.34};\\ s_{f}=540\frac{t_{a}^{0.4}w_{a}{}^{1.7}}{f_{t,a}};G_{f}=243t_{a}^{0.4}w_{a}{}^{0.437}; \end{array}$	CFRP plate ($E_{c}=165\mathrm{GPa}$); linear adhesives Sikadur-30	The surfaces of steel plates were sandblasted and cleaned with acetone	Cohesive failure; delamination within CFRP plate
	Equation (3)	$\begin{array}{l} s_{1}=2.9\frac{\tau_{f}}{G_{a}}t_{a}^{0.34};\tau_{f}=0.9f_{t,a};\\ s_{2}=180\frac{t_{a}^{0.4}w_{a}{}^{1.7}}{f_{t,a}}+0.85\frac{t_{a}^{0.34}f_{t,a}}{G_{a}};\\ s_{f}=360\frac{t_{a}^{0.4}w_{a}{}^{1.7}}{f_{t,a}}+1.7\frac{t_{a}^{0.34}f_{t,a}}{G_{a}};\\ G_{f}=243t_{a}^{0.4}w_{a}{}^{0.437}; \end{array}$	CFRP plate ($E_{c}=165\mathrm{GPa}$); non-linear adhesive Araldite 2015		Cohesive failure
Pang et al., 2021 [29]	Equation (1)	$\begin{array}{l} \tau_{f}=0.544\tau_{c}^{1.21};\;s_{1}=\frac{1.51\tau_{c}^{1.21}}{G_{a}}t_{a}^{0.378};\\ s_{f}=\frac{149t_{a}^{0.196}w_{c}{}^{1.22}}{\tau_{c}^{1.21}};G_{f}=40.5t_{a}^{0.196}w_{c}{}^{1.22}; \end{array}$	CFRP plate ($E_{c}=165\mathrm{GPa}$); linear adhesives Sikadur-30 (CN) and sticky steel resin R	The surfaces of steel plates were sandblasted and the CFRP plates were lightly abraded with fine sandpaper	Delamination within CFRP plate

**Table 2:** Material properties.

Material	Specification	Thickness/mm	Tensile Modulus of Elasticity/GPa	Yield Strength/MPa	Tensile Strength /MPa	Elongation at Break/%
CFRP plate	CFP-1-514	1.4	165 ^a^	N/A	2400 ^a^	1.61 ^a^
Adhesive	Sikadur-30CN	N/A	5.3	N/A	41.75	1.13
Steel plate	Q235B	10.75 ^b^	181.9	275.6	421.18	20.78

Notes: ^a^ According to the manufacturer’s instructions. ^b^ Thickness of steel plate cut from the flange of the uncorroded H beams.

**Table 3:** Characteristic value of bond properties.

Specimen No.	Cd /Month	ξ /%	$S_{z}$ /um	$t_{a}$ /mm	$\tau_{f}$ /mm	$t_{\mathrm{eff}}$ /mm	$s_{1}$ /mm	α	$G_{f}$ /N·mm^−1^	$s_{f}$ /mm
C0-B5-T1	0	0	157.35	0.41	21.440	0.4887	0.023	0.678	0.9084	0.080
C0-B5-T2				1.44	22.486	1.5187	0.024	0.912	1.0556	0.093
C0-B5-T3				1.42	23.538	1.4987	0.031	0.830	1.2400	0.103
C0-B5-T4				1.94	18.134	2.0187	0.029	0.963	0.9795	0.107
C3-B5-T1	3	5.08	1301.7	0.37	21.324	1.0209	0.024	0.905	1.0582	0.098
C3-B5-T2				1.10	19.310	1.7509	0.027	0.849	1.2623	0.129
C3-B5-T3				1.44	19.584	2.0909	0.026	0.923	1.1126	0.113
C3-B5-T4				1.41	19.627	2.0609	0.032	0.978	1.1122	0.113
C4-B5-T1	4	6.15	845.95	0.70	22.864	1.1230	0.027	0.709	1.0338	0.086
C6-B5-T	6	7.92	874.75	0.24	20.357	0.6774	0.018	0.542	0.9622	0.089
C6-B5-T2				1.01	22.207	1.4474	0.021	0.815	1.2372	0.109
C6-B5-T3				1.30	20.901	1.7374	0.023	0.964	1.3789	0.132
C6-B5-T4				2.10	18.986	2.5374	0.031	0.999	1.1935	0.126
C8-B5-T1	8	10.07	937.7	0.42	21.454	0.8889	0.019	0.709	0.8863	0.079
C8-B5-T2				0.80	21.376	1.2689	0.025	1.001	1.1731	0.110
C8-B5-T3				0.87	20.588	1.3389	0.031	1.007	1.1804	0.115
C8-B5-T4				1.72	22.415	2.1889	0.035	0.988	0.9775	0.087
C12-B5-T1	12	15.02	993.3	0.70	18.605	1.1967	0.025	0.805	1.0659	0.112

Notes: *C*_d_ and *ξ* are the corrosion duration and weight loss rates of corroded steel plates, respectively, *S*_z_ is the maximum height of the corroded steel surface, $t_{a}$ is the adhesive thickness, $\tau_{f}$ is the maximum bond resistance, $t_{\mathrm{eff}}$ is the effective adhesive thickness, $s_{1}$ is the relative slip corresponding to the peak interfacial shear stress, *α* is the fitting parameter for the ascending segment of the bond-slip curve, $G_{f}$ is the fracture energy, $s_{f}$ is the maximum relative slip.

## Data Availability

Not applicable.
